# Space Flight Calcium: Implications for Astronaut Health, Spacecraft Operations, and Earth

**DOI:** 10.3390/nu4122047

**Published:** 2012-12-18

**Authors:** Scott M. Smith, Torin McCoy, Daniel Gazda, Jennifer L. L. Morgan, Martina Heer, Sara R. Zwart

**Affiliations:** 1 Human Health and Performance Directorate, NASA Lyndon B. Johnson Space Center, Houston, TX 77058, USA; E-Mail: torin.mccoy-1@nasa.gov; 2 Wyle Science, Technology & Engineering Group, Houston, TX 77058, USA; E-Mail: daniel.b.gazda@nasa.gov; 3 Oak Ridge Associated Universities/NASA Post-Doctoral Fellow, NASA Lyndon B. Johnson Space Center, Houston, TX 77058, USA; E-Mail: jennifer.morgan@nasa.gov; 4 Profil, 41460 Neuss, Germany; E-Mail: martina.heer@profil.com; 5 University of Bonn, 53115 Bonn, Germany; 6 Division of Space Life Sciences, Universities Space Research Association, Houston, TX 77058, USA; E-Mail: sara.zwart-1@nasa.gov

**Keywords:** bed rest, bone, calcium, collagen crosslinks, dual-energy X-ray absorptiometry, space flight

## Abstract

The space flight environment is known to induce bone loss and, subsequently, calcium loss. The longer the mission, generally the more bone and calcium are lost. This review provides a history of bone and calcium studies related to space flight and highlights issues related to calcium excretion that the space program must consider so that urine can be recycled. It also discusses a novel technique using natural stable isotopes of calcium that will be helpful in the future to determine calcium and bone balance during space flight.

## 1. Introduction

Bone and calcium metabolism have been a concern for space travelers, literally since before human space flight was a reality. A little more than a half century after the first human space flight, we have improved our understanding of the effects of space flight on calcium and bone, but we still have much to learn. We review here the current state of knowledge and describe ongoing studies, including application of the findings to space exploration and implications for the general population.

## 2. Bone Loss

Space flight-induced bone and calcium loss have been documented for decades, and have been the subject of many reviews [1,2,3,4,5]. There have also been several evaluations of ground-based analogs, including the most common, bed rest [6,7]. Bed rest is a viable model of space flight-induced bone loss, producing metabolic changes and bone loss that are qualitatively similar to those brought on by space flight but have a smaller magnitude. Bone loss in bed rest is about half of that observed in space flight [8,9].

Documentation of negative calcium balance and increased calcium excretion during space flight first came from Gemini and Apollo missions of the 1960s and early 1970s [10,11], but bed rest studies documenting negative calcium balance go back even further, with initial documentation in the 1940s [12]. Although urinary and fecal calcium excretion were shown clearly to increase on short-duration space flights (1–2 weeks of flight), not until the longer Skylab missions were flown (1973–1974) were actual changes in bone observed using densitometry [13,14].

In the 1990s the Russian space station Mir provided a platform for long-duration studies on changes in bone during space flight. Dual-energy X-ray absorptiometry (DXA) scans on astronauts and cosmonauts began to better characterize and quantify bone loss during space flight [2,15,16]. Although considerable site-to-site variability exists, along with crewmember-to-crewmember variability, in general, a 1.0%–1.5% loss of bone mineral density occurred per month of space flight [2]. Bone biochemistry studies in the 1990s also expanded significantly, largely fueled by the identification of collagen crosslinks as markers that could be used to assess bone resorption, and development of immunoassays to measure these crosslinks along with markers of bone formation. Novel techniques are being developed to assess overall bone balance using stable isotopes of calcium. These techniques will be beneficial because they are noninvasive (only a urine sample is needed), might be possible to do during flight, and will give a picture of overall bone balance instead of resorption or formation alone. These techniques will be discussed in detail below.

Early flight experiments with animals pointed to a decrease in bone formation as the metabolic key to bone loss, whereas human studies (both space flight and bed rest) clearly pointed to an increase in bone resorption, with bone formation being either unchanged or slightly decreased [17,18,19,20,21,22]. Further research documented that the discrepancy between the animal and human data was not caused by a problem with the animal model itself, but rather by an issue with the age of the animals used in those early flight studies. In young rats, bone formation is suppressed during space flight. In mature rats, bone resorption predominates and little change is seen in bone formation [23], as evidenced in hindlimb-suspension studies. These differences in mechanism of bone metabolism must be considered when interpreting results from space flight studies. Young animals are commonly used in research, and provide an added benefit for space flight studies in that more animals may be flown in the limited space available for animal research on these missions. Although any model has limitations, and these need to be carefully accounted for, animal studies do provide the ability to do more extensive research on mechanisms of bone loss.

In 2000, the first crews took up residence on the International Space Station (ISS), marking the beginning of what many hope will be a permanent human presence off the planet. One of the novel aspects of the ISS was the inclusion of resistance exercise equipment. Details of these efforts will be described below, but although initial evaluations were disappointing [24], possibly because of altered kinematics [25], recent evidence illustrates the potential of proper nutrition and exercise regimens to prevent whole-body and regional loss of bone mineral density [26] during extended space flight. 

## 3. Calcium Isotopes and Relevance for Bone Turnover

Analytical techniques to assess bone health, bone loss, and bone metabolism continue to evolve with technology. Although densitometry techniques (such as DXA and quantitative computerized tomography) provide valuable assessment of specific bones, these techniques detect only relatively large changes in bone, and it takes several months for changes of this magnitude to occur. Studying calcium requires either intensive balance studies or tracer kinetic studies. Bone biochemical markers can provide more rapid assessments of changes in bone formation or resorption, but assessing the relative association of these two factors has not been possible to date, and thus it is difficult to assess net changes in bone calcium content.

**Figure 1:**
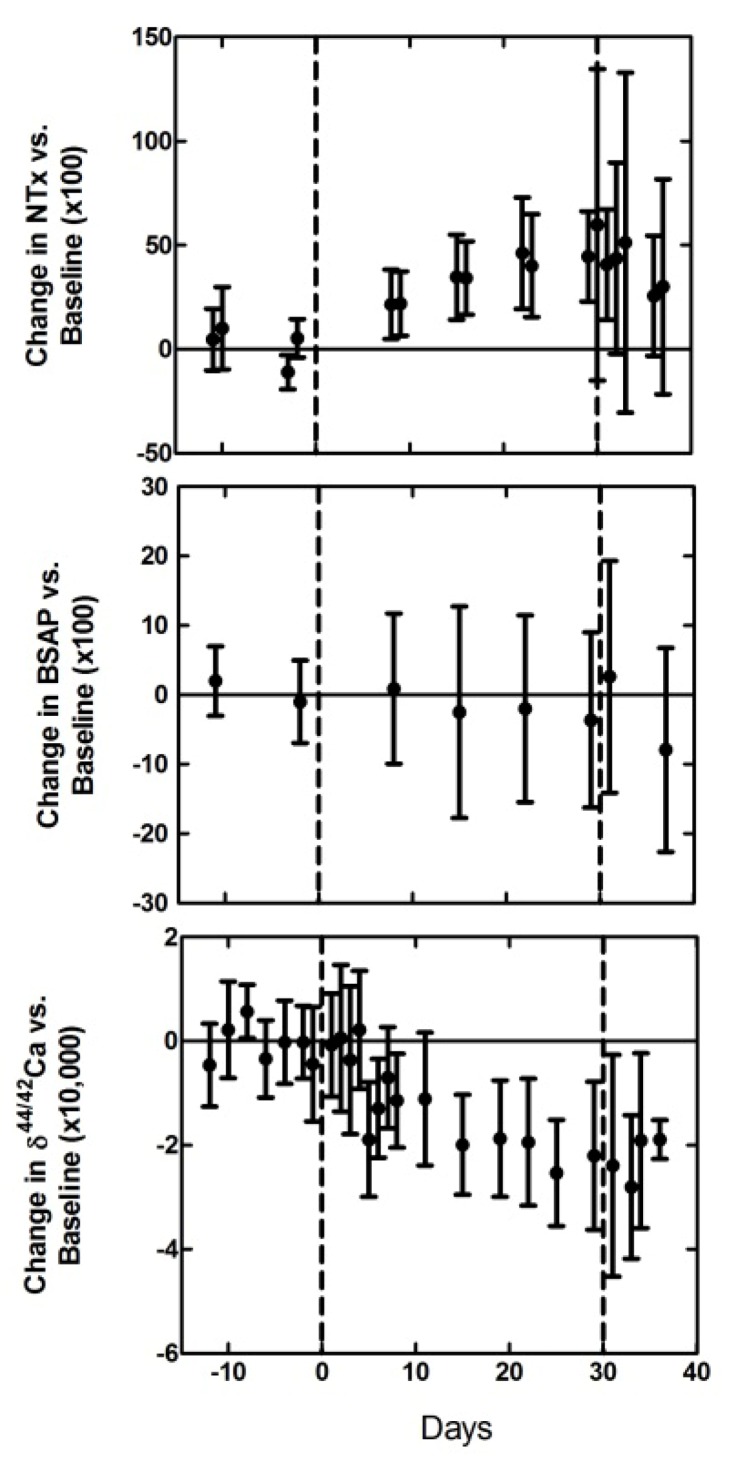
Variations in bone biochemical markers *N*-telopeptide (NTX, top panel) and bone-specific alkaline phosphatase (BSAP, middle panel), along with calcium isotopes (bottom panel), during and after bed rest (days 0 to 40). Percent changes were calculated as the difference between the measured value at each time point and the average of the pre-bed rest values (baseline, days to left of day 0) for that individual. All values are mean ± SD. The calcium isotopes shift in a direction consistent with bone loss after just 7 days of bed rest and track the signal observed in NTX while BSAP remains unchanged. Note: This figure is adapted with permission from [27], Copyright © 2012 National Academy of Sciences. Data are from 12 subjects.

A new technique to rapidly detect and quantitatively predict changes in bone mineral balance (the ratio of bone formation to resorption) has recently been validated in a bed rest model [27]. Changes in bone mineral balance as a result of bed rest can be detected by measuring the ratios of stable calcium isotopes in urine from individuals who have not received any stable isotope tracers [28]. This calcium isotope biomarker is based on natural, biologically induced variations. These variations are a result of the 6 naturally occurring calcium isotopes (^40^Ca, ^42^Ca, ^43^Ca, ^44^Ca, ^46^Ca, and ^48^Ca) reacting at different rates depending on their mass [29]. In general, isotopic selectivity can occur in all elements with multiple stable isotopes, and has been measured in light elements such as hydrogen, oxygen, carbon, and nitrogen for many decades. These variations arise because the vibrational frequencies of any chemical bonds are a function of the masses of the constituent nuclides. As a result, more energy is required to break bonds formed from heavy isotopes than for the same bond with a lighter isotope. In chemical reactions that do attain equilibrium (which are rare in biology), heavier isotopes are preferentially concentrated in the strongest chemical bonding environments. In chemical reactions that do not attain equilibrium (e.g., kinetic reactions), the bonds incorporating lighter isotopes usually react more quickly than those incorporating heavier isotopes. Kinetic processes like diffusion, evaporation, and precipitation, preferentially select lighter isotopes because they move faster than heavy isotopes [30,31]. In soft tissue (e.g., blood, urine), variations in the calcium isotope composition exist because bone formation depletes soft tissue of lighter calcium isotopes most likely as a result of isotope selectivity during osteoblast-induced calcium precipitation. Bone resorption releases that isotopically light calcium back into soft tissue. Therefore, when bone is being resorbed, as is the case during bed rest, the urinary calcium isotope abundance shifts toward lighter values (*i.e.*, −δ^44/42^Ca; more ^42^Ca and less ^44^Ca relative to baseline). Applying this technique to a bed rest study, it was shown that the calcium isotope ratio shifted in a direction consistent with bone loss after just 7 days of bed rest, long before detectable changes in bone density occur. Consistent with this interpretation, the calcium isotope variation accompanied changes observed in *N*-telopeptide, while bone-specific alkaline phosphatase, a bone-formation biomarker, was unchanged (Figure 1) [27]. 

**Figure 2:**
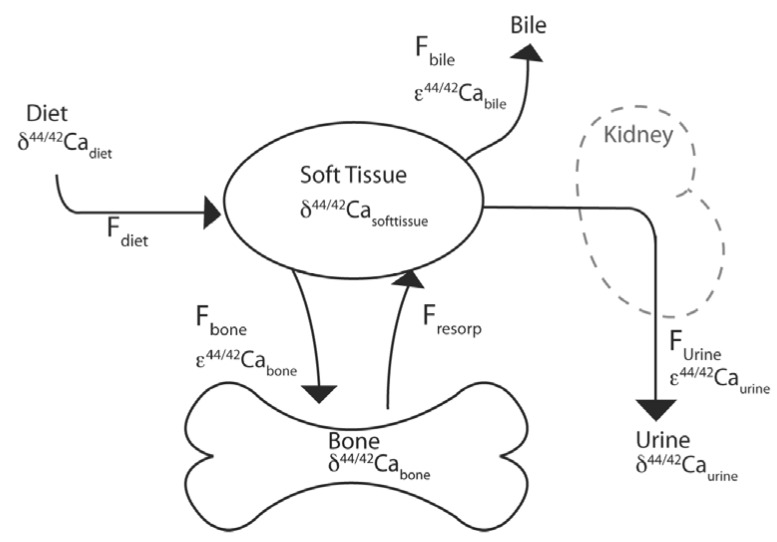
Schematic of model pools and fluxes used to quantify bone loss. F_diet_ is the flux of calcium absorbed from diet, F_bone_ and F_resorp_ are the bone formation and resorption fluxes, and F_urine_ and F_bile_ are the net excretion fluxes of calcium through urine and bile respectively. δ^44/42^Ca_soft_ and δ^44/42^Ca_bone_ are the Ca isotopic compositions of the soft tissue and bone pools, δ^44/42^Ca_diet_ and δ^44/42^Ca_urine_ are the isotopic compositions of the fluxes of Ca absorbed from diet and excreted in urine, and ε^44/42^Ca_bone, _ε^44/42^Ca_urine, _and ε^44/42^Ca_bile_ are the isotopic variations associated with forming bone, urine, and bile, respectively, from the soft tissue pool. Note: This figure is adapted with permission from [27], Copyright © 2012 National Academy of Sciences.

As the relationship between calcium isotopes and bone mineral balance is well established based on the isotope selectivity principles described above and preliminary work measuring the offset between soft tissue and bone [32,33], this relationship can be used to quantitatively translate the changes in the calcium isotope ratio in urine to changes in bone mineral density using a simple model (Figure 2) [27]. Using this model it was estimated that subjects lost 0.25% ± 0.07% (1 SD) of their bone mass from day 7 to day 30 of bed rest [27]. This rate of loss extrapolates to a loss of 1.36% ± 0.38% of skeletal mass over 119 days, which is equivalent, within error, to bone loss rates determined by DXA scans in long-term (119-day) bed rest studies [34]. 

A preliminary study examined the calcium isotope shift in bed rest subjects undergoing three different treatments (untreated bed rest, the bisphosphonate alendronate, and intense resistance exercise) [35,36]. In this study there were significant differences in isotope response: the control group’s calcium isotope composition shifted in a direction consistent with bone resorption, while the alendronate and resistance exercise groups’ calcium isotope composition shifted in a manner suggesting maintained and slightly increased bone formation, respectively [28]. 

Given the rapid signal observed using calcium isotope measurements and the potential to quantitatively assess bone loss, this technique is ideally suited for space flight studies in which changes in bone formation and resorption are not only being altered by space flight itself but are being manipulated by various countermeasures.

## 4. Dietary Calcium

When bone loss is considered, calcium intake is an obvious initial concern. Skylab crews consumed an average of 894 ± 142 (SD) mg calcium/day [8] while participating in carefully planned and executed metabolic balance studies over the entire course of their missions (28, 59, and 84 days) [14,37].

Based on reviews of available information, a decision was made requiring that the ISS food system shall provide 1000–1200 mg calcium per day [8], similar to Earth-based recommendations. Proximate analysis of the foods provided in the “standard” menu revealed that calcium content of the ISS menu averages 1020 ± 109 mg calcium/day [8]. 

Although the crews self-select meals while they are on board the ISS, dietary intake estimates using a food frequency questionnaire designed for space flight documented actual intakes of calcium during flight that were in the same range as in the “standard” menu, 1068 ± 384 mg calcium/day in one report [38], and 912 ± 229 and 1025 ± 309 mg calcium/day in another [26]. Thus, intakes typically are close to or meeting planned calcium intake during flight, which is similar to the Earth-based recommended intake.

## 5. Calcium Absorption, Metabolism, and Excretion

In space flight and bed rest models, changes in bone biochemistry occur rapidly after humans enter the altered environment. This is hypothesized to be related to a loss of bone in response to unloading, and the release of calcium from bone, which suppresses parathyroid hormone. This suppression of parathyroid hormone in turn is associated with a drop in circulating 1,25-dihydroxyvitamin D and a decrease in calcium absorption. 

In space flight studies, serum total and ionized calcium are tightly regulated, and do not manifest as consistent measurable changes during flight [21,26,39,40]. Parathyroid hormone decreases during flight [26], although because of the small numbers of subjects, this change is not always statistically significant [18,22]. Circulating concentrations of the active form of vitamin D—1,25-dihydroxyvitamin D—are decreased during flight (vitamin D stores will be addressed below). Calcium absorption has been shown by calcium tracer kinetic studies to be decreased during flight [21,22,41]. This decreased absorption has also been indirectly evidenced through increased fecal calcium during flight [10,14,37]. Urinary calcium excretion is also increased during flight (this will be addressed in greater detail below).

In bed rest, findings generally similar to those from space flight have been documented: unchanged total circulating calcium [42,43,44,45], unchanged [44,45] or slightly increased ionized calcium [42], decreased parathyroid hormone [44,45,46,47,48] and 1,25-dihydroxyvitamin D concentrations [42,45,46], decreased calcium absorption [42], and increased urinary [42,43,45,46,47,48,49] and fecal calcium [42]. As mentioned earlier, although the biochemical and physiological changes in bed rest are similar to those observed in space flight, the magnitudes of the changes are less in the ground-based analog. As with any area of research, particularly research with human subjects, not all studies reveal the same effects on all analytes. For example, some studies have shown increased serum calcium levels (within normal limits) [46,47]. Not all studies have shown decreases in circulating parathyroid hormone concentrations during bed rest; some have shown trends that were not statistically significant [47,49], others have shown clearly unchanged concentrations [42,43]. Many factors are likely to lead to these discrepancies, including study design and controls; subject age, gender, and pre-study fitness levels; laboratory analytical methods; and inherent lab-to-lab variability. Despite these discrepancies, bed rest remains the best analog of space flight with regard to bone and calcium metabolism.

## 6. Bone Loss Countermeasures

Along with the identification of space flight-induced bone loss, came a significant effort to find a means to counteract this loss. Many countermeasures have been proposed and studied, including physical, nutritional, and pharmacological measures. As with virtually all space flight research, ground testing is required to document proof of concept before a countermeasure is implemented on a space mission. Thus, numerous bed rest and related analog studies have been done in which bone countermeasures were evaluated.

Physical countermeasures tested in bed rest studies have included exercise, vibration, and centrifugation. Resistance exercise has shown the most promise in combating bone loss that results from skeletal unloading. Heavy resistance exercise (such as weight-lifting) results in increased bone formation but has little or no effect on resorption, while maintaining bone mineral density [36]. When heavy resistance exercise was combined with an aerobic type of exercise countermeasure (treadmill exercise within a lower-body negative pressure device), the effect was similar, although a striking dose-response relationship of the effect was observed [47]. That is, when subjects performed resistance exercise only every other day (3–4 days/week), the increase in bone formation was half of what it was in subjects who performed resistance exercise 6 days per week.

Low-level vibration seemed promising as a bone loss countermeasure judging by a pilot study with human subjects and animal model data. However, although in bed rest vibration had some beneficial effects on bone marrow and vertebral disks [50,51,52], it did not protect bone mineral density as was initially hoped [53]. Although vibration of higher intensity or a combination of vibration and resistance exercise produced more encouraging results with bone and muscle [48,54,55,56,57,58], some concerns have been raised about potential risks of repetitive-motion injuries. 

Space flight-induced bone loss is clearly related to a lack of the gravity found on Earth. Preliminary studies have been conducted to evaluate the counteraction of bone loss by artificial gravity, produced either by centrifugation [59,60] or by simply standing or walking for specified time periods during the bed rest phase of bed rest studies. Although standing or walking for 2–4 h daily during bed rest reduced hypercalciuria [61], an hour of centrifugation daily failed to have a positive effect on bone health [44,62]. Many have proposed that subjects exercise during centrifugation, and although this has proven beneficial for the muscle and cardiovascular systems [63], clear effects on bone have yet to be documented. Artificial gravity should clearly, at some point, have an effect on bone health, but the ability to provide this on spacecraft will be an engineering challenge.

Nutritional countermeasures also have the potential to mitigate bone loss. One potential nutritional countermeasure is mitigation of low-level metabolic acidosis that can result from metabolism of dietary components, including excess amounts of sulfur-containing amino acids and sodium chloride. Decreasing the intake of sulfur-containing amino acids and decreasing the intake of sodium chloride have been documented to mitigate bone loss in bed rest studies, and both of these nutritional countermeasures are being tested on the ISS. In one study the impact of altering the ratio of animal protein to potassium in the diet (not from supplements) is being examined, using animal protein as a proxy for sulfur-containing amino acids and potassium as an estimate for organic base precursor salts (carbonate, *etc.*) in the diet [64,65,66,67]. The literature contains many conflicting studies of the effects of protein on bone, but bed rest (and space flight) provides a unique situation, in which generally healthy individuals are placed in an environment that induces bone loss. The negative effect of protein on bone seems to occur during bed rest (but not during an ambulatory control phase), and is more pronounced with greater duration of bed rest [64,65,66,67]. Negative effects of protein on bone have also been found in studies of individuals with inadequate intake of calcium, vitamin D, protein, or other nutrients, or individuals with mobility challenges. However, in many studies of healthy, ambulatory, well-nourished individuals, no effect (or at least no negative effect) of protein on bone was found [68]. This is an area where space flight studies might help to bridge gaps in the literature, in documenting that neither case is “right” or “wrong,” but rather that the conditions of the experiments in essence provide different models for the effect of protein on bone metabolism.

Most of the offered space food up to now is rather high in sodium, mainly sodium chloride, content. This results in a high dietary sodium intake for residents on the ISS, whose average intake is more than 5000 mg of sodium per day, with individual intakes exceeding 12,000 mg/day [8,9,38,69,70]. In the general population, high sodium chloride intake induces increased urinary calcium excretion [71,72,73,74,75,76,77] and renal stone risk [78]. Controlled studies in humans have shown that high sodium chloride intake may lead to low-grade metabolic acidosis [79,80]. As the resorbing cells, osteoclasts, need an acidic environment to be activated [81,82,83], the acidosis that results from high sodium chloride intake might cause increased bone resorption as shown by bone resorption markers [80]. This effect is exacerbated when bone-resorbing mechanisms are already activated because of the immobilization in bed rest [84]. Bone formation, on the other hand, does not seem to be affected by acidosis, as shown by bone formation markers [84]. How much high sodium chloride intake affects bone turnover during space flight is currently being examined on the ISS in controlled studies, by comparing bone turnover in phases of low and high sodium intake. To what extent the increased amount of dietary sodium chloride is responsible for the loss of bone mass in space flight is not known. However, taking into account the extremely high sodium intake in the daily diet of astronauts [8,9,38,69], it is very likely that bone loss can be decreased by lowering daily sodium chloride intake. As a consequence of that, and further undesirable effects of high sodium chloride intake in space flight, NASA has made a tremendous effort to decrease the salt content in the American space food products.

Omega-3 fatty acid intake has been shown to mitigate bone resorption in bed rest [85]. This was supported by cell culture studies in which eicosapentaenoic acid suppressed NFκB activation in osteoclasts [85]. Controlled studies in humans and animals also support a protective role of fish and omega-3 fatty acids in bone health [86,87,88,89,90]. Controlled studies during space flight have not yet been possible, but a somewhat crude negative association between fish intake and bone loss has been established [85].

Vitamin D is a concern for space travelers, in part because their dietary sources of vitamin D are insufficient, and in part because they lack ultraviolet light exposure [8]. Vitamin D stores decline during flight if supplemental intake of this vitamin is inadequate [22,38]. Ground analog studies have been conducted in Antarctica [91,92], where crews who stay during the winter also get little or no ultraviolet light exposure. Recent studies have documented that supplementation with 800 IU vitamin D/day will maintain vitamin D stores in astronauts on 6-month space missions [26]. Although vitamin D is likely not a countermeasure for space flight-induced bone loss, vitamin D deficiency will surely exacerbate the problem.

Studies with other nutrients have also been attempted, with the aim of providing higher intake of calcium [43] or vitamin K [93,94], but to date these have not provided enough convincing ground-based data to warrant further investigation of calcium or vitamin K intake [95]. 

Pharmacologic studies focusing on bisphosphonates have been conducted in bed rest (and related human and animal models) with several forms of these antiresorptive drugs [35,96,97,98,99]. Initial in-flight testing with alendronate was conducted recently [100]. Initial results seem promising, but concerns over side effects arise with any pharmaceutical agent.

Exogenous testosterone is also often proposed as a potential countermeasure for bone and muscle loss of space flight. This idea is based on data from a 1993 Space Shuttle flight, with 4 subjects and one data point for each subject [101]. It is noteworthy that these 4 subjects were also consuming about half of their energy requirement in the days before data collection [69]. Detailed analysis of data from 15 crewmembers on long-duration flights to the ISS found no change in circulating testosterone during flight in well-nourished crewmembers [102]. Similar findings were also reported in bed rest subjects [102].

Combinations of countermeasures, including heavy resistance exercise and good nutritional intakes, have recently been associated with maintenance of bone mineral density during space flight. ISS crewmembers consuming an adequate diet (maintaining intake of energy and other nutrients, including maintaining optimal vitamin D status) and exercising with an enhanced suite of exercise equipment, including treadmill, cycle, and a high-load resistance exercise device were able to maintain their whole-body and regional bone mineral density at preflight levels [26]. This regimen led to an increase in bone formation without suppressing the space flight-induced increase in bone resorption, and thus questions and concerns remain about bone strength. Nonetheless, after more than a half century of space travel and many proposed countermeasures that were unable to mitigate bone mineral density loss, this is a significant step forward.

## 7. Urinary Calcium and Renal Stone Risk

One of the immediate concerns about increased urinary calcium during space flight is the increased risk of renal stone formation. This increased risk has been documented during both short- and long-duration space missions [103,104,105], as well as in bed rest [106,107,108,109,110]. Given that the occurrence of a kidney stone on orbit would likely result in the medical evacuation of the affected crewmember (and likely end the mission for all crewmembers, given the nature of spacecraft), the likelihood of a kidney stone occurring has warranted a fair amount of attention. Many factors can increase or decrease renal stone risk, but increased fluid intake is clearly the easiest, and a proven effective, means to reduce this risk [111]. Space-flight studies suggest that increased fluid intake is a viable countermeasure after flight, when stone risk remains increased relative to preflight levels [112]. However, in some cases urine chemistry changes may indicate that additional in-flight countermeasures are required [113]. One such potential countermeasure is potassium citrate, which has an alkalinizing effect on urine and thus will reduce stone-forming potential [114].

## 8. Urinary Calcium and Spacecraft Water Reclamation

Renal stone risk and bone loss are well known to be medical concerns for space flight, and recently urinary calcium precipitation became a concern for operation of the ISS Program for an entirely different reason. In this section, we will walk through the process undertaken by NASA’s Nutritional Biochemistry Laboratory and Water and Food Analytical Laboratory to make recommendations to the ISS Program related to urinary calcium concentration and fluid intake, and we will provide the in-flight urine data that were used to make these decisions. 

In late 2008 NASA deployed the Urine Processor Assembly (UPA), a system designed to reclaim water from urine. Reclaiming water from urine is a significant step toward closing the life-support loop on the ISS and significantly reduces the resupply requirement for this critical resource. 

The UPA works on the principle of reduced-pressure distillation. Preserved urine is collected in a metal bellows tank that feeds a fixed-volume recirculation loop. As water is removed from the preserved urine, additional urine flows from the bellows tank to maintain the volume in the recirculation loop. The loop also contains a brine tank that is emptied at the end of each concentration cycle. The amount of water recovered during each concentration cycle is calculated as a percentage of the total amount of urine added to the recirculation loop. The initial goal for the UPA was to recover 85% of the water in urine. This corresponds to 232 L of water for a 41-L brine tank and 119 L of water for a 21-L brine tank. The brine that is collected during each concentration cycle is discarded in the non-reusable resupply vehicles that deliver cargo to the ISS. 

**Figure 3:**
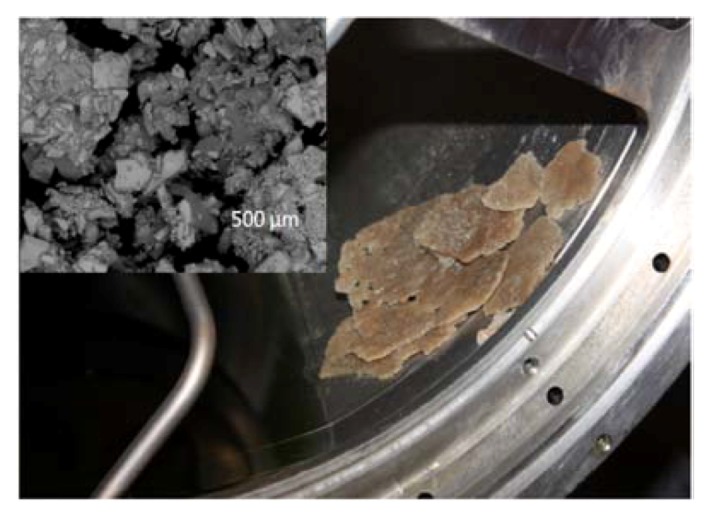
Photograph of calcium sulfate precipitates formed inside the Urine Processor Assembly (UPA) distillation assembly on the International Space Station (ISS), and scanning electron micrograph (inset) of precipitates formed during UPA ground tests. Photo and micrograph courtesy of the NASA Marshall Space Flight Center Environmental Control and Life Support (ECLS) Project.

After initial deployment and checkout of the UPA, and several months of normal operations, a hard failure occurred in the system. This failure was later determined to be the result of precipitation in the distillation assembly and recirculation loop. Several brine samples and hardware components were returned as part of the failure investigation, and after extensive ground analyses the precipitate was identified as calcium sulfate. The primary source of the sulfate was sulfuric acid, which is one component of the preservative added to urine when it is collected in the waste hygiene compartment (toilet). The preservative is added to inhibit biological activity and prevent ammonia formation while the urine is stored before it is processed. The sulfate combined with the calcium in urine to form the precipitate, which coated the surface of the distillation assembly (Figure 3).

An extensive review of urinary calcium excretion during space flight was conducted after the failure occurred, because preflight testing of the UPA had shown no evidence of precipitation. Evaluation of existing in-flight urinary calcium data shed new light on the cause of the UPA failure and, by extension, the risk of renal stone formation during space flight. Data were initially evaluated in 2010 and then the updated data set was reanalyzed in 2012. By then, the data set had expanded to include samples from 10 additional subjects (2–5 in-flight samples for each subject). The intent of revisiting the data was to determine if the findings of the initial assessment were still valid. We report both data sets here and elaborate on the impact of the assessments on water reclamation on the ISS and on evaluation of renal stone risk. 

**Figure 4:**
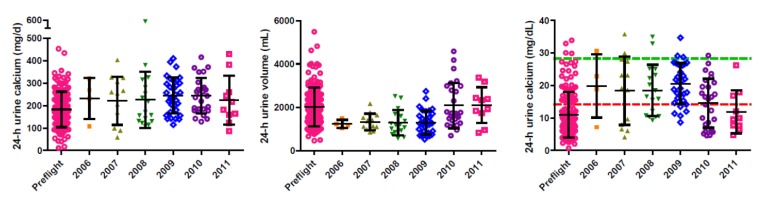
Urinary calcium excretion (left), urine volume (middle), and urinary calcium concentration (right) before and during flight, by year of collection on the ISS. Each symbol represents a 24-h urine collection; 23 crewmembers may have each provided up to five 24-h collections during flight, and at least 4 before flight. Within data sets, the horizontal lines represent the mean ± 1 SD. The green dashed line in the right panel represents a calcium concentration of 28.3 mg/dL, the expected calcium precipitation point for UPA water recovery at 70%, and the red dashed line represents calcium concentrations of 14.2 mg/dL, the expected calcium precipitation point for UPA water recovery at 85%. A subset of these data, in a different form, have been published, along with details of urine collection procedures [26].

Water recovery in the UPA is limited by the concentration of calcium in the urine, and not the total amount of calcium excreted per day. For this reason, total urine volume was a key variable in the assessments. Figure 4 shows urinary calcium excretion, urine volume, and urinary calcium concentration data before and during flight, separated by year of collection. Independent of time, urinary calcium excretion is increased during flight (as noted above), and urine volume is decreased. This combination of effects results in higher urinary calcium concentrations during space flight. The red lines in the right panel reflect the calculated values above which precipitation would be expected when recovering 85% of the water. As shown in Figure 4, given the number of data points above the lines in 2009, the initial precipitation event that occurred on orbit should have been expected.

After the initial data review and analysis in 2010, a decision was made to reduce the water recovery rate to 65%, which significantly reduced the amount of water recovered during each concentration cycle. Eventually, the recovery rate was increased to 70%, but that was still much lower than the initially targeted 85%. When the data were reevaluated in 2012, the expanded data set indicated that the average of all combined urinary calcium concentrations had dropped by 8% relative to the average of data available in 2010. This was largely due to the fact that the average urinary calcium concentrations for the 10 additional subjects were 18% lower than those for the first 13 subjects (Figure 4). When daily urinary calcium excretion and total urine volumes were examined, this difference in calcium concentration was found to be almost entirely the result of increased urine volumes (Figure 4).

**Figure 5:**
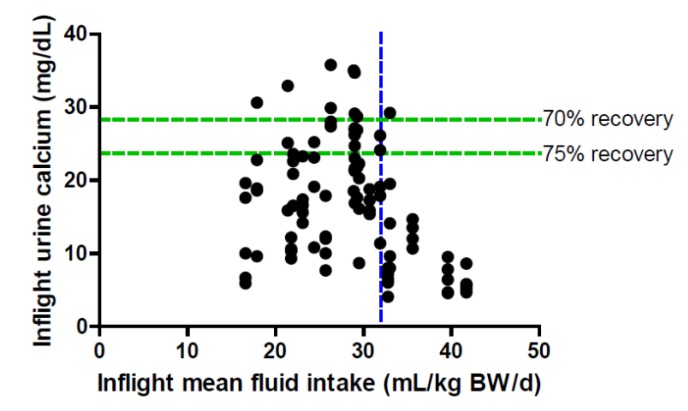
In-flight fluid intake and associated in-flight urinary calcium concentration of crewmembers on the ISS. Each symbol represents a 24-h urine collection; individual crewmembers may have provided up to five 24-h collections during flight, and at least 4 before flight. Dashed horizontal lines reflect urinary calcium concentrations above which calcium precipitation would be expected for UPA water recoveries at 70% and 75%.

Data from in-flight food frequency questionnaires (FFQs) allowed us to compare fluid intake over time; the average fluid intake increased from 1904 ± 414 mL/day in crews that launched in 2006–2007 to 2284 ± 184 mL/day in crews that launched in 2010. This increased fluid intake was consistent with the observed higher urine volumes and may be attributed to a number of factors, including education of the crews on the importance of fluid intake. The need for adequate fluid intake was emphasized in preflight briefings with crews after the initial precipitation event. The UPA served as a perfect analog for the human kidney to illustrate the potential for formation of precipitates (renal stones). Another contributing factor could be launching and installation of the U.S. Potable Water Dispenser in early 2009. This provided a second dispensing point for potable water on the ISS and simplified access to water. Importantly, the chance that the more recent crews simply happened to habitually drink more water cannot be ignored. Regardless of the cause, in evaluating these data, it was determined that consumption of fluid at >32 mL/kg body weight was associated with urinary calcium concentrations that would allow recovery of water at 75% with minimal risk of precipitation (Figure 5) and also reduce the risk of renal stone formation.

For the 23 crewmembers included in the data set, 32 mL/kg body weight equals an average of 2.5 L/day of fluid, the amount of water that the water recovery system on the ISS is designed to provide for crewmembers. Thus, this level of water consumption would not be considered extraordinary or even atypical. The fluid intake requirement for ISS crewmembers is 1.0–1.5 mL/kcal, but not less than 2000 mL/day [8].

**Figure 6:**
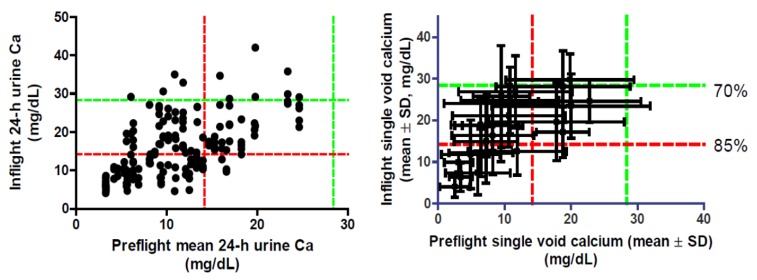
Relationships between preflight and in-flight urinary calcium concentrations. Left panel: Each symbol represents a 24-h urine collection, and on the x axis individual crewmember preflight data were averaged, with individual in-flight data points shown on the graph. Right panel: Each point reflects the average of all individual urine voids, and the error bars reflect one standard deviation of the data before flight (horizontal) or during flight (vertical). The green dashed lines represent calcium concentrations of 28.3 mg/dL, the expected calcium precipitation point for UPA water recovery at 70%, and the red dashed lines represent calcium concentrations of 14.2 mg/dL, the expected calcium precipitation point for UPA water recovery at 85%.

From an ISS Program point of view, it would be ideal if one could predict in-flight urinary calcium concentrations on the basis of preflight data. This would allow personalized consultations with individual crewmembers to minimize their health risks during flight and tailor water recovery to reduce precipitation risks for a given crew complement. To this end, the available data were evaluated from two perspectives: using 24-h pools (Figure 6, left panel), and using single-void urinary calcium concentration data (Figure 6, right panel) to determine whether a predictable relationship existed between preflight and in-flight urinary calcium concentrations. These data suggest that although variability is substantial, there does seem to be a relationship.

**Figure 7:**
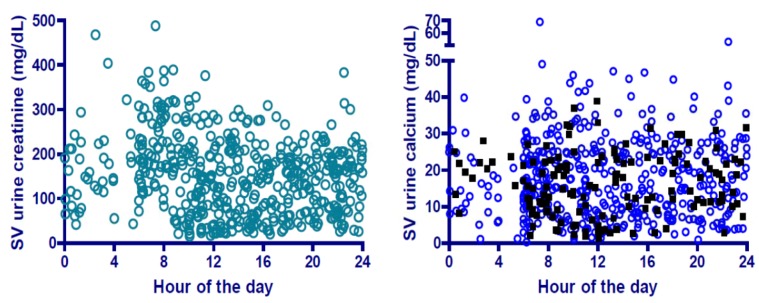
Creatinine (left panel) and calcium (right panel) concentration in single-void (SV) urine samples, by time of day when samples were collected. Some ISS crewmembers were participating in an experiment on bisphosphonate (alendronate) use as a bone loss countermeasure. These data are shown with filled symbols, but did not have a significant effect on calcium concentration.

In the attempt to understand the nature of urinary calcium and operational aspects of this system, many facets were examined. Data were evaluated to determine if urinary calcium concentrations changed over the course of a mission, but no significant relationship was observed. That is, urinary calcium concentration was essentially the same earlier and later in the 6-month mission. Single-void data were also evaluated to assess whether excluding the first morning void from water recovery would eliminate higher average calcium concentrations in urine, but again, somewhat surprisingly, this was found not to be the case (Figure 7). This was true for subjects participating in a study in which 70 mg of alendronate was administered once weekly during flight, and also was true for those not taking alendronate (Figure 7).

Results from the expanded data set and documented efforts to educate crews on the importance of adequate fluid intake led the ISS Program in 2012 to make the decision to increase water recovery by the UPA from 70% to 74%–75%. This decision required the periodic reevaluation of urinary calcium concentrations to assess whether or not the trend for increased fluid intake and urine output is being maintained. Increasing the water recovery percentage in the UPA will reclaim an additional 60–80 L of water per year. When one considers the cost of launching 60–80 kg of water, the 4%–5% increase in water recovery yields a significant cost savings. An additional benefit of increasing the water recovery rate in the UPA is the reduction in crew time associated with changing the brine tank. Crew time is arguably the most valuable resource on the ISS, and running the UPA at 74%–75% recovery is estimated to save 6–8 h of crew time each year.

## 9. Conclusions

Many health risks are associated with alterations in bone and calcium metabolism during space flight, extending from short-term risk of renal stone formation to long-term bone loss. Understanding calcium metabolism during space flight, and developing methods to counteract and detect bone loss, will be critical for exploration beyond low Earth orbit. As briefly reviewed here, these studies and findings will have multiple applications to space travel (including impacts on non-human spacecraft systems), and will have implications for the medical and scientific communities here on Earth as well.

## References

[1] Sibonga J.D., Cavanagh P.R., Lang T.F., LeBlanc A.D., Schneider V.S., Shackelford L.C., Smith S.M., Vico L. (2008). Adaptation of the skeletal system during long-duration spaceflight. Clin. Rev. Bone Miner. Metab..

[2] LeBlanc A., Schneider V., Shackelford L., West S., Oganov V., Bakulin A., Voronin L. (2000). Bone mineral and lean tissue loss after long duration space flight. J. Musculoskelet. Neuronal Interact..

[3] Alexandre C., Vico L. (2011). Pathophysiology of bone loss in disuse osteoporosis. Joint Bone Spine.

[4] Whedon G.D., Rambaut P.C. (2006). Effects of long-duration spaceflight on calcium metabolism: Review of human studies from Skylab to the present. Acta Astronaut..

[5] Heer M., Kamps N., Biener C., Korr C., Boerger A., Zittermann A., Stehle P., Drummer C. (1999). Calcium metabolism in microgravity. Eur. J. Med. Res..

[6] LeBlanc A.D., Spector E.R., Evans H.J., Sibonga J.D. (2007). Skeletal responses to space flight and the bed rest analog: A review. J. Musculoskelet. Neuronal Interact..

[7] Pavy-Le Traon A., Heer M., Narici M.V., Rittweger J., Vernikos J. (2007). From space to Earth: Advances in human physiology from 20 years of bed rest studies (1986–2006). Eur. J. Appl. Physiol..

[8] Smith S.M., Zwart S.R., Kloeris V., Heer M. (2009). Nutritional Biochemistry of Space Flight.

[9] Smith S.M., Zwart S.R. (2008). Nutritional biochemistry of spaceflight. Adv. Clin. Chem..

[10] Lutwak L., Whedon G.D., Lachance P.A., Reid J.M., Lipscomb H.S. (1969). Mineral, electrolyte and nitrogen balance studies of the Gemini-VII fourteen-day orbital space flight. J. Clin. Endocrinol. Metab..

[11] Rambaut P.C., Leach C.S., Johnson P.C. (1975). Calcium and phosphorus change of the Apollo 17 crew members. Nutr. Metab..

[12] Deitrick J.E., Whedon G.D., Shorr E. (1948). Effects of immobilization upon various metabolic and physiologic functions of normal men. Am. J. Med..

[13] Whedon G., Lutwak L., Rambaut P., Whittle M., Leach C., Reid J., Smith M. (1976). Effect of weightlessness on mineral metabolism; metabolic studies on Skylab orbital flights. Calcif. Tissue Res..

[14] Whedon G.D., Lutwak L., Rambaut P.C., Whittle M.W., Smith M.C., Reid J., Leach C., Stadler C.R., Sanford D.D., Johnston R.S., Dietlein L.F. (1977). Mineral and Nitrogen Metabolic Studies, Experiment M071. Biomedical Results from Skylab (NASA Sp-377).

[15] Oganov V.S., Grigoriev A.I., Voronin L.I., Rakhmanov A.S., Bakulin A.V., Schneider V.S., LeBlanc A.D. (1992). Bone mineral density in cosmonauts after flights lasting 4.5–6 months on the Mir orbital station. Aviakosm. Ekolog. Med..

[16] Vico L., Collet P., Guignandon A., Lafage-Proust M.H., Thomas T., Rehaillia M., Alexandre C. (2000). Effects of long-term microgravity exposure on cancellous and cortical weight-bearing bones of cosmonauts. Lancet.

[17] Collet P., Uebelhart D., Vico L., Moro L., Hartmann D., Roth M., Alexandre C. (1997). Effects of 1- and 6-month spaceflight on bone mass and biochemistry in two humans. Bone.

[18] Caillot-Augusseau A., Lafage-Proust M.H., Soler C., Pernod J., Dubois F., Alexandre C. (1998). Bone formation and resorption biological markers in cosmonauts during and after a 180-day space flight (Euromir 95). Clin. Chem..

[19] Smith S.M., Nillen J.L., LeBlanc A., Lipton A., Demers L.M., Lane H.W., Leach C.S. (1998). Collagen cross-link excretion during space flight and bed rest. J. Clin. Endocrinol. Metab..

[20] Lueken S.A., Arnaud S.B., Taylor A.K., Baylink D.J. (1993). Changes in markers of bone formation and resorption in a bed rest model of weightlessness. J. Bone Miner. Res..

[21] Smith S.M., Wastney M.E., Morukov B.V., Larina I.M., Nyquist L.E., Abrams S.A., Taran E.N., Shih C.Y., Nillen J.L., Davis-Street J.E. (1999). Calcium metabolism before, during, and after a 3-mo spaceflight: kinetic and biochemical changes. Am. J. Physiol..

[22] Smith S.M., Wastney M.E., O’Brien K.O., Morukov B.V., Larina I.M., Abrams S.A., Davis-Street J.E., Oganov V., Shackelford L.C. (2005). Bone markers, calcium metabolism, and calcium kinetics during extended-duration space flight on the Mir space station. J. Bone Miner. Res..

[23] Smith B.J., King J.B., Lucas E.A., Akhter M.P., Arjmandi B.H., Stoecker B.J. (2002). Skeletal unloading and dietary copper depletion are detrimental to bone quality of mature rats. J. Nutr..

[24] Lang T., LeBlanc A., Evans H., Lu Y., Genant H., Yu A. (2004). Cortical and trabecular bone mineral loss from the spine and hip in long-duration spaceflight. J. Bone Miner. Res..

[25] Pierre M.C., Genc K.O., Litow M., Humphreys B., Rice A.J., Maender C.C., Cavanagh P.R. (2006). Comparison of knee motion on Earth and in space: An observational study. J. Neuroengineering Rehabil..

[26] Smith S.M., Heer M.A., Shackelford L., Sibonga J.D., Ploutz-Snyder L., Zwart S.R. (2012). Benefits for bone from resistance exercise and nutrition in long-duration spaceflight: Evidence from biochemistry and densitometry. J. Bone Miner. Res..

[27] Morgan J.L., Skulan J.L., Gordon G.W., Romaniello S.J., Smith S.M., Anbar A.D. (2012). Rapidly assessing changes in bone mineral balance using natural stable calcium isotopes. Proc. Natl. Acad. Sci. USA.

[28] Skulan J., Bullen T., Anbar A.D., Puzas J.E., Shackelford L., LeBlanc A., Smith S.M. (2007). Natural calcium isotopic composition of urine as a marker of bone mineral balance. Clin. Chem..

[29] Russell W.A., Papanastassiou D.A., Tombrello T.A. (1978). Ca isotope fractionation on earth and other solar-system materials. Geochim. Cosmochim. Acta.

[30] DePaolo D.J., Johnson C., Beard B., Albarede F. (2004). Geochemistry of Non-Traditional Stable Isotopes.

[31] Criss R.E. (1999). Principles of Stable Isotope Distribution.

[32] Morgan J.L., Gordon G.W., Arrua R.C., Skulan J.L., Anbar A.D., Bullen T.D. (2011). High-precision measurement of variations in calcium isotope ratios in urine by multiple collector inductively coupled plasma mass spectrometry. Anal. Chem..

[33] Skulan J.L., DePaolo D.J. (1999). Calcium isotope fractionation between soft and mineralized tissue as a monitor of calcium use in vertebrates. Proc. Natl. Acad. Sci. USA.

[34] Spector E.R., Smith S.M., Sibonga J.D. (2009). Skeletal effects of long-duration head-down bed rest. Aviat. Space Environ. Med..

[35] LeBlanc A.D., Driscol T.B., Shackelford L.C., Evans H.J., Rianon N.J., Smith S.M., Feeback D.L., Lai D. (2002). Alendronate as an effective countermeasure to disuse induced bone loss. J. Musculoskelet. Neuronal Interact..

[36] Shackelford L.C., LeBlanc A.D., Driscoll T.B., Evans H.J., Rianon N.J., Smith S.M., Spector E., Feeback D.L., Lai D. (2004). Resistance exercise as a countermeasure to disuse-induced bone loss. J. Appl. Physiol..

[37] Whedon G.D., Lutwak L., Reid J., Rambaut P., Whittle M., Smith M., Leach C. (1975). Mineral and nitrogen balance study: Results of metabolic observations on Skylab II 28-day orbital mission. Acta Astronaut..

[38] Smith S.M., Zwart S.R., Block G., Rice B.L., Davis-Street J.E. (2005). The nutritional status of astronauts is altered after long-term space flight aboard the International Space Station. J. Nutr..

[39] Caillot-Augusseau A., Lafage-Proust M.H., Margaillan P., Vergely N., Faure S., Paillet S., Lang F., Alexandre C., Estour B. (2000). Weight gain reverses bone turnover and restores circadian variation of bone resorption in anorexic patients. Clin. Endocrinol. (Oxf.).

[40] Smith S.M., Davis-Street J.E., Fontenot T.B., Lane H.W. (1997). Assessment of a portable clinical blood analyzer during space flight. Clin. Chem..

[41] Zittermann A., Heer M., Caillot-Augusso A., Rettberg P., Scheld K., Drummer C., Alexandre C., Horneck G., Vorobiev D. (2000). Microgravity inhibits intestinal calcium absorption as shown by a stable strontium test. Eur. J. Clin. Invest..

[42] LeBlanc A., Schneider V., Spector E., Evans H., Rowe R., Lane H., Demers L., Lipton A. (1995). Calcium absorption, endogenous excretion, and endocrine changes during and after long-term bed rest. Bone.

[43] Baecker N., Frings-Meuthen P., Smith S.M., Heer M. (2010). Short-term high dietary calcium intake during bedrest has no effect on markers of bone turnover in healthy men. Nutrition.

[44] Smith S.M., Zwart S.R., Heer M.A., Baecker N., Evans H.J., Feiveson A.H., Shackelford L.C., Leblanc A.D. (2009). Effects of artificial gravity during bed rest on bone metabolism in humans. J. Appl. Physiol..

[45] Morgan J.L., Zwart S.R., Heer M., Ploutz-Snyder R., Ericson K., Smith S.M. (2012). Bone metabolism and nutritional status during 30-day head-down tilt bed rest. J. Appl. Physiol..

[46] Smith S.M., Davis-Street J.E., Fesperman J.V., Calkins D.S., Bawa M., Macias B.R., Meyer R.S., Hargens A.R. (2003). Evaluation of treadmill exercise in a lower body negative pressure chamber as a countermeasure for weightlessness-induced bone loss: A bed rest study with identical twins. J. Bone Miner. Res..

[47] Smith S.M., Zwart S.R., Heer M., Lee S.M.C., Baecker N., Meuche S., Macias B.R., Shackelford L.C., Schneider S., Hargens A.R. (2008). WISE-2005: Supine treadmill exercise within lower body negative pressure and flywheel resistive exercise as a countermeasure to bed rest-induced bone loss in women during 60-day simulated microgravity. Bone.

[48] Armbrecht G., Belavy D.L., Gast U., Bongrazio M., Touby F., Beller G., Roth H.J., Perschel F.H., Rittweger J., Felsenberg D. (2010). Resistive vibration exercise attenuates bone and muscle atrophy in 56 days of bed rest: Biochemical markers of bone metabolism. Osteoporos. Int..

[49] Baecker N., Tomic A., Mika C., Gotzmann A., Platen P., Gerzer R., Heer M. (2003). Bone resorption is induced on the second day of bed rest: results of a controlled crossover trial. J. Appl. Physiol..

[50] Holguin N., Uzer G., Chiang F.P., Rubin C., Judex S. (2011). Brief daily exposure to low-intensity vibration mitigates the degradation of the intervertebral disc in a frequency-specific manner. J. Appl. Physiol..

[51] Ozcivici E., Luu Y.K., Rubin C.T., Judex S. (2010). Low-level vibrations retain bone marrow’s osteogenic potential and augment recovery of trabecular bone during reambulation. PLoS One.

[52] Holguin N., Muir J., Rubin C., Judex S. (2009). Short applications of very low-magnitude vibrations attenuate expansion of the intervertebral disc during extended bed rest. Spine J..

[53] Baecker N., Frings-Meuthen P., Heer M., Mester J., Liphardt A.M. (2012). Effects of vibration training on bone metabolism: Results from a short-term bed rest study. Eur. J. Appl. Physiol..

[54] Belavy D.L., Armbrecht G., Gast U., Richardson C.A., Hides J.A., Felsenberg D. (2010). Countermeasures against lumbar spine deconditioning in prolonged bed rest: Resistive exercise with and without whole body vibration. J. Appl. Physiol..

[55] Belavy D.L., Beller G., Armbrecht G., Perschel F.H., Fitzner R., Bock O., Borst H., Degner C., Gast U., Felsenberg D. (2010). Evidence for an additional effect of whole-body vibration above resistive exercise alone in preventing bone loss during prolonged bed rest. Osteoporos. Int..

[56] Belavy D.L., Miokovic T., Armbrecht G., Rittweger J., Felsenberg D. (2009). Resistive vibration exercise reduces lower limb muscle atrophy during 56-day bed-rest. J. Musculoskelet. Neuronal Interact..

[57] Belavy D.L., Wilson S.J., Armbrecht G., Rittweger J., Felsenberg D., Richardson C.A. (2012). Resistive vibration exercise during bed-rest reduces motor control changes in the lumbo-pelvic musculature. J. Electromyogr. Kinesiol..

[58] Blottner D., Salanova M., Puttmann B., Schiffl G., Felsenberg D., Buehring B., Rittweger J. (2006). Human skeletal muscle structure and function preserved by vibration muscle exercise following 55 days of bed rest. Eur. J. Appl. Physiol..

[59] Warren L.E., Reinertson R., Camacho M.E., Paloski W.H. (2007). Implementation of the NASA Artificial Gravity Bed Rest Pilot Study. J. Gravit. Physiol..

[60] Clément G., Bukley A.P. (2007). Artificial Gravity.

[61] Vernikos J. (1997). Artificial gravity intermittent centrifugation as a space flight countermeasure. J. Gravit. Physiol..

[62] Zwart S.R., Crawford G.E., Gillman P.L., Kala G., Rodgers A.S., Rogers A., Inniss A.M., Rice B.L., Ericson K., Coburn S. (2009). Effects of 21 days of bed rest, with or without artificial gravity, on nutritional status of humans. J. Appl. Physiol..

[63] Yang Y., Baker M., Graf S., Larson J., Caiozzo V.J. (2007). Hypergravity resistance exercise: The use of artificial gravity as potential countermeasure to microgravity. J. Appl. Physiol..

[64] Zwart S.R., Davis-Street J.E., Paddon-Jones D., Ferrando A.A., Wolfe R.R., Smith S.M. (2005). Amino acid supplementation alters bone metabolism during simulated weightlessness. J. Appl. Physiol..

[65] Zwart S.R., Hargens A.R., Lee S.M., Macias B.R., Watenpaugh D.E., Tse K., Smith S.M. (2007). Lower body negative pressure treadmill exercise as a countermeasure for bed rest-induced bone loss in female identical twins. Bone.

[66] Zwart S.R., Hargens A.R., Smith S.M. (2004). The ratio of animal protein intake to potassium intake is a predictor of bone resorption in space flight analogues and in ambulatory subjects. Am. J. Clin. Nutr..

[67] Zwart S.R., Smith S.M. (2005). The impact of space flight on the human skeletal system and potential nutritional countermeasures. Int. SportMed J..

[68] Whiting S.J., Boyle J.L., Thompson A., Mirwald R.L., Faulkner R.A. (2002). Dietary protein, phosphorus and potassium are beneficial to bone mineral density in adult men consuming adequate dietary calcium. J. Am. Coll. Nutr..

[69] Heer M., Boerger A., Kamps N., Mika C., Korr C., Drummer C. (2000). Nutrient supply during recent European missions. Pflugers Arch..

[70] Smith S.M., Davis-Street J., Rice B.L., Lane H.W. (1997). Nutrition in space. Nutr. Today.

[71] Goulding A. (1981). Fasting urinary sodium/creatinine in relation to calcium/creatinine and hydroxyproline/creatinine in a general population of women. N. Z. Med. J..

[72] Arnaud S.B., Wolinsky I., Fung P., Vernikos J. (2000). Dietary salt and urinary calcium excretion in a human bed rest spaceflight model. Aviat. Space Environ. Med..

[73] Nordin B.E., Need A.G., Morris H.A., Horowitz M. (1993). The nature and significance of the relationship between urinary sodium and urinary calcium in women. J. Nutr..

[74] Carbone L.D., Bush A.J., Barrow K.D., Kang A.H. (2003). The relationship of sodium intake to calcium and sodium excretion and bone mineral density of the hip in postmenopausal African-American and Caucasian women. J. Bone Miner. Metab..

[75] Chan A.Y., Poon P., Chan E.L., Fung S.L., Swaminathan R. (1993). The effect of high sodium intake on bone mineral content in rats fed a normal calcium or a low calcium diet. Osteoporos. Int..

[76] Chan E.L., Ho C.S., MacDonald D., Ho S.C., Chan T.Y., Swaminathan R.  (1992). Interrelationships between urinary sodium, calcium, hydroxyproline and serum PTH in healthy subjects. Acta Endocrinol. (Copenh.).

[77] Massey L.K., Whiting S.J. (1996). Dietary salt, urinary calcium, and bone loss. J. Bone Miner. Res..

[78] Massey L.K., Whiting S.J. (1995). Dietary salt, urinary calcium, and stone risk. Nutr. Rev..

[79] Heer M., Frings-Meuthen P., Titze J., Boschmann M., Frisch S., Baecker N., Beck L. (2009). Increasing sodium intake from a previous low or high intake affects water, electrolyte and acid-base differently. Br. J. Nutr..

[80] Frings-Meuthen P., Baecker N., Heer M. (2008). Low-grade metabolic acidosis may be the cause of sodium chloride-induced exaggerated bone resorption. J. Bone Miner. Res..

[81] Arnett T. (2003). Regulation of bone cell function by acid-base balance. Proc. Nutr. Soc..

[82] Arnett T.R. (2008). Extracellular pH regulates bone cell function. J. Nutr..

[83] Arnett T.R., Dempster D.W. (1986). Effect of pH on bone resorption by rat osteoclasts* in vitro*. Endocrinology.

[84] Frings-Meuthen P., Buehlmeier J., Baecker N., Stehle P., Fimmers R., May F., Kluge G., Heer M. (2011). High sodium chloride intake exacerbates immobilization-induced bone resorption and protein losses. J. Appl. Physiol..

[85] Zwart S.R., Pierson D., Mehta S., Gonda S., Smith S.M. (2010). Capacity of omega-3 fatty acids or eicosapentaenoic acid to counteract weightlessness-induced bone loss by inhibiting NF-kappaB activation: From cells to bed rest to astronauts. J. Bone Miner. Res..

[86] Griel A.E., Kris-Etherton P.M., Hilpert K.F., Zhao G., West S.G., Corwin R.L. (2007). An increase in dietary *n*-3 fatty acids decreases a marker of bone resorption in humans. Nutr. J..

[87] Bonnet N., Ferrari S.L. (2011). Effects of long-term supplementation with omega-3 fatty acids on longitudinal changes in bone mass and microstructure in mice. J. Nutr. Biochem..

[88] Rousseau J.H., Kleppinger A., Kenny A.M. (2009). Self-reported dietary intake of omega-3 fatty acids and association with bone and lower extremity function. J. Am. Geriatr. Soc..

[89] Tartibian B., Hajizadeh Maleki B., Kanaley J., Sadeghi K. (2011). Long-term aerobic exercise and omega-3 supplementation modulate osteoporosis through inflammatory mechanisms in post-menopausal women: A randomized, repeated measures study. Nutr. Metab..

[90] Watkins B.A., Li Y., Lippman H.E., Feng S. (2003). Modulatory effect of omega-3 polyunsaturated fatty acids on osteoblast function and bone metabolism. Prostaglandins Leukot. Essent. Fatty Acids.

[91] Zwart S.R., Mehta S.K., Ploutz-Snyder R., Bourbeau Y., Locke J.P., Pierson D.L., Smith S.M. (2011). Response to vitamin D supplementation during Antarctic winter is related to BMI, and supplementation can mitigate Epstein-Barr virus reactivation. J. Nutr..

[92] Smith S.M., Gardner K.K., Locke J., Zwart S.R. (2009). Vitamin D supplementation during Antarctic winter. Am. J. Clin. Nutr..

[93] Vermeer C., Wolf J., Craciun A.M., Knapen M.H. (1998). Bone markers during a 6-month space flight: Effects of vitamin K supplementation. J. Gravit. Physiol..

[94] Vermeer C., Wolf J., Knapen M.H. (1997). Microgravity-induced changes of bone markers: Effects of vitamin K-supplementation. Bone.

[95] Zwart S.R., Booth S.L., Peterson J.W., Wang Z., Smith S.M. (2011). Vitamin K status in spaceflight and ground-based models of spaceflight. J. Bone Miner. Res..

[96] Lockwood D.R., Vogel J.M., Schneider V.S., Hulley S.B. (1975). Effect of the diphosphonate EHDP on bone mineral metabolism during prolonged bed rest. J. Clin. Endocrinol. Metab..

[97] Rittweger J., Frost H.M., Schiessl H., Ohshima H., Alkner B., Tesch P., Felsenberg D. (2005). Muscle atrophy and bone loss after 90 days’ bed rest and the effects of flywheel resistive exercise and pamidronate: Results from the LTBR study. Bone.

[98] Schultheis L., Ruff C.B., Rastogi S., Bloomfield S., Hogan H.A., Fedarko N., Thierry-Palmer M., Ruiz J., Bauss F., Shapiro J.R. (2000). Disuse bone loss in hindquarter suspended rats: Partial weightbearing, exercise and ibandronate treatment as countermeasures. J. Gravit. Physiol..

[99] Shapiro J., Smith B., Beck T., Ballard P., Dapthary M., Brintzenhofeszoc K., Caminis J. (2007). Treatment with zoledronic acid ameliorates negative geometric changes in the proximal femur following acute spinal cord injury. Calcif. Tissue Int..

[100] LeBlanc A., Matsumoto T., Jones J., Shapiro J., Lang T., Shackelford L., Smith S.M., Evans H., Spector E., Ploutz-Snyder L. (2012). Bisphosphonates as a supplement to exercise to protect bone during long-duration spaceflight. Osteoporos. Int..

[101] Strollo F., Strollo G., More M., Bollanti L., Ciarmatori A., Longo E., Quintiliani R., Mambro A., Mangrossa N., Ferretti C. (1998). Hormonal adaptation to real and simulated microgravity. J. Gravit. Physiol..

[102] Smith S.M., Heer M., Wang Z., Huntoon C.L., Zwart S.R. (2012). Long-duration space flight and bed rest effects on testosterone and other steroids. J. Clin. Endocrinol. Metab..

[103] Whitson P.A., Pietrzyk R.A., Morukov B.V., Sams C.F. (2001). The risk of renal stone formation during and after long duration space flight. Nephron.

[104] Whitson P.A., Pietrzyk R.A., Pak C.Y. (1997). Renal stone risk assessment during Space Shuttle flights. J. Urol..

[105] Okada A., Ichikawa J., Tozawa K. (2011). Kidney stone formation during space flight and long-term bed rest. Clin. Calcium.

[106] Hwang T.I., Hill K., Schneider V., Pak C.Y. (1988). Effect of prolonged bedrest on the propensity for renal stone formation. J. Clin. Endocrinol. Metab..

[107] Monga M., Macias B., Groppo E., Kostelec M., Hargens A. (2006). Renal stone risk in a simulated microgravity environment: Impact of treadmill exercise with lower body negative pressure. J. Urol..

[108] Okada A., Ohshima H., Itoh Y., Yasui T., Tozawa K., Kohri K. (2008). Risk of renal stone formation induced by long-term bed rest could be decreased by premedication with bisphosphonate and increased by resistive exercise. Int. J. Urol..

[109] Watanabe Y., Ohshima H., Mizuno K., Sekiguchi C., Fukunaga M., Kohri K., Rittweger J., Felsenberg D., Matsumoto T., Nakamura T. (2004). Intravenous pamidronate prevents femoral bone loss and renal stone formation during 90-day bed rest. J. Bone Miner. Res..

[110] Zerwekh J.E., Odvina C.V., Wuermser L.A., Pak C.Y. (2007). Reduction of renal stone risk by potassium-magnesium citrate during 5 weeks of bed rest. J. Urol..

[111] Pak C.Y., Sakhaee K., Crowther C., Brinkley L. (1980). Evidence justifying a high fluid intake in treatment of nephrolithiasis. Ann. Intern. Med..

[112] Whitson P., Pietrzyk R., Pak C., Cintron N. (1993). Alterations in renal stone risk factors after space flight. J. Urol..

[113] Whitson P.A., Pietrzyk R.A., Sams C.F. (2001). Urine volume and its effects on renal stone risk in astronauts. Aviat. Space Environ. Med..

[114] Whitson P.A., Pietrzyk R.A., Jones J.A., Nelman-Gonzalez M., Hudson E.K., Sams C.F. (2009). Effect of potassium citrate therapy on the risk of renal stone formation during spaceflight. J. Urol..

